# Glial Cells and Their Function in the Adult Brain: A Journey through the History of Their Ablation

**DOI:** 10.3389/fncel.2017.00024

**Published:** 2017-02-13

**Authors:** Sarah Jäkel, Leda Dimou

**Affiliations:** ^1^Physiological Genomics, Biomedical Center, Ludwig-Maximilians UniversityMunich, Germany; ^2^MRC Centre for Regenerative Medicine, University of EdinburghEdinburgh, UK; ^3^Munich Cluster for Systems NeurologyMunich, Germany; ^4^Molecular and Translational Neuroscience, Department of Neurology, University of UlmUlm, Germany

**Keywords:** cell ablation, astrocytes, microglia, NG2-glia, oligodendrocytes, brain function

## Abstract

Glial cells, consisting of microglia, astrocytes, and oligodendrocyte lineage cells as their major components, constitute a large fraction of the mammalian brain. Originally considered as purely non-functional glue for neurons, decades of research have highlighted the importance as well as further functions of glial cells. Although many aspects of these cells are well characterized nowadays, the functions of the different glial populations in the brain under both physiological and pathological conditions remain, at least to a certain extent, unresolved. To tackle these important questions, a broad range of depletion approaches have been developed in which microglia, astrocytes, or oligodendrocyte lineage cells (i.e., NG2-glia and oligodendrocytes) are specifically ablated from the adult brain network with a subsequent analysis of the consequences. As the different glial populations are very heterogeneous, it is imperative to specifically ablate single cell populations instead of inducing cell death in all glial cells in general. Thanks to modern genetic manipulation methods, the approaches can now directly be targeted to the cell type of interest making the ablation more specific compared to general cell ablation approaches that have been used earlier on. In this review, we will give a detailed summary on different glial ablation studies, focusing on the adult mouse central nervous system and the functional readouts. We will also provide an outlook on how these approaches could be further exploited in the future.

## Introduction

Glial cells were first identified by the 19th century’s leading neuroscientists including Rudolf Virchow, Santiago Ramón y Cajal and Pío del Río-Hortega. At that time, glia were suggested to solely function as so-called “Nervenkitt” (the German word for nerve glue). This is also reflected in the name “glial cell” derived from the ancient Greek word “glía” meaning “glue” in English. With time, scientists started to speculate about additional possible roles of these cells. Although many studies have been performed to specify these further roles, the full properties of glial cells remain unresolved. Moreover, glial cells are anything but a minor cellular fraction, as they constitute – depending on the mammalian species – between 33 and 66% of the total brain mass (Azevedo et al., 2009; Herculano-Houzel, 2014). Recent findings have made it clear that glial cells are more than just mere “Nervenkitt”. The total glial cell population can be subdivided into four major groups: (1) microglia, (2) astrocytes, (3) oligodendrocytes, and (4) their progenitors NG2-glia. This review will focus on the research of the past decades addressing the role of these four major glial cell types in regard to the function of the adult brain.

The analysis of cellular function can be addressed through a range of different experimental measures. One common technique involves the depletion of mature brain cells that are already integrated in an established network *in vivo*. During the last two decades, various ways have been developed to achieve this goal. Initially, cytotoxic substances, such as ethidium bromide (EtBr) that are lethal for cells in general (including neurons) were used (Yajima and Suzuki, 1979). To specifically target and subsequently ablate cycling cells, the application of high doses of X-irradiation (Kalderon and Fuks, 1996; Chari and Blakemore, 2002) or of the mitotic blocker arabinofuranosyl cytidine (AraC, Doetsch et al., 1999) to the tissue of interest has also proven to be successful. These general ablation approaches, which are still in use today, were likely favored due to the simplicity of application and due to the lack of more specific alternatives. X-irradiation as well as the application of AraC is not cell type specific, as all cycling cells in the area (X-irradiation) or tissue (AraC) of interest undergo induced cell death. Therefore these approaches are not ideal to identify the function of a specific cell type. The same is true for EtBr, as it induces general unrepairable DNA damage leading to cell death (Yajima and Suzuki, 1979; Johnson et al., 2003). The use of such general approaches has decreased, as drugs can have effects in other tissues that lead to secondary damage or even changes in the cells of interest. As genetic manipulation has become easier and more accessible within the recent years, cell ablation techniques have also improved. Nowadays, most ablation approaches make use of a cell type or a region-specific promoter that is coupled with a “suicide” gene, resulting in depletion of distinct cell types. Suicide genes typically encode either a toxin, an enzyme that converts a pro-drug into a toxic agent, or an essential protein for the specific cell type leading to apoptotic cell death specifically in the cells of interest. The main advantage of these approaches is that they have few side effects for surrounding cells or other tissues.

Application of these ablation methods have already helped us to better understand the functions of all four major glial cell types in the adult brain. This review will summarize and discuss the major findings of ablation studies performed during the last few decades.

## Microglia

### Microglia Ablation under Healthy Conditions

In a very simplified view, microglia are the immunocompetent and phagocytic cells of the nervous system. Although they are part of the brain’s glia, they do not originate from the ectodermal tissue like all other glial cells, but from yolk-sac progenitors that only populate the brain during development (reviewed in Kim and de Vellis, 2005; Kettenmann et al., 2011). Microglia have been shown to cover a huge volume of the adult brain parenchyma, with individual non-overlapping domains constantly sensing the environment through rapid movements of their fine filopodia, which react to any kind of insult (Nimmerjahn et al., 2005; Cronk and Kipnis, 2013). As microglia in the brain have the same origin and express many common cellular markers with peripheral macrophages/monocytes, it has proven difficult to only ablate one but not the other cell type (Silver et al., 2015). Additionally, macrophages are able to invade the brain upon injury or any other disturbance of the blood brain barrier (BBB), meaning that the roles of microglia and macrophages have been difficult to disentangle (Pineau et al., 2010). Several of the microglia ablation studies tried to address the issue of how these cells maintain their homeostasis in the adult healthy brain. Microglia could either derive from a brain intrinsic stem cell source or from a peripheral derived progenitor originating from the same developmental source that infiltrates the brain at some stage and contributes to the microglia population. From these studies, it seemed that at least under physiological conditions the two populations remain separated (see below). In addition, there is the question about other roles played by microglia besides surveying the healthy tissue, as microglia have been shown to be major players in synaptic pruning during development (reviewed in Wu et al., 2015) and in synaptic modulation both in normal as well as under pathological conditions (Bessis et al., 2007; Hong et al., 2016a,b; Vasek et al., 2016). Further microglia ablation studies could therefore provide a potential tool to describe yet unknown microglia-associated functions in adult brain physiology.

Although several studies have depleted myeloid cells – including microglia – during development by inserting deleterious mutations in macrophage-specific genes (reviewed in Waisman et al., 2015), this review will focus only on microglia ablation studies performed in the adult brain.

To successfully ablate adult microglia, pharmacological and genetic strategies have been developed (for summary and details see **Table 1**). As a pharmacological approach, the use of the systemically administered drug PLX3397, which specifically targets Colony-stimulating factor 1 receptor (CSF-1R) signaling, has been well established. CSF-1R is uniquely expressed on myeloid cells including brain resident microglia, making only these cells susceptible to death (Elmore et al., 2014). The use of clodronate liposomes (CLs) is a second pharmacological way to specifically deplete phagocytic cells, including microglia (van Rooijen et al., 1990). After the phagocytic cells take up the liposomal particles and release the encapsulated, toxic clodronate they undergo apoptosis. As a genetic approach, the diphtheria toxin (DT) or its subunit A (DTA), originally derived from the bacterium *Corynebacterium diphtheriae*, is a widely used suicide gene that has already been applied in ablation approaches. Its mode of action is the cytosolic inhibition of cellular protein synthesis, leading to cell death (Honjo et al., 1968). The DT-dependent ablation system can be used in two ways: (1) direct tamoxifen-inducible expression of the DT(A) in the target cells or (2) cell type-specific expression of the diphtheria toxin receptor (DTR) in combination with the systemic application of DT(A), such that only cells carrying the receptor are susceptible to death. Independent of methodology (pharmacological or genetic), all of these approaches achieve fast and robust microglia death in the brain, ranging from 80 to >99% depending on the treatment and the region of interest (Parkhurst et al., 2013; Elmore et al., 2014, 2015; Bruttger et al., 2015; Torres et al., 2016).

**Table 1:** Microglial ablation approaches under healthy and pathological conditions.

Cell type	Condition	Method	Efficiency (%)	Physiological effect	Reference
Microglia	Health	PLX3397	>99	Fast repopulation, no negative outcome	Elmore et al., 2014, 2015
		PLX3397, clodronate liposomes (CLs)	∼80	Transient and reversible changes in spatial memory and social behavior	Torres et al., 2016
		CX_3_CR1^CreER^:iDTR mouse line	>90	Loss of synapse formation, negative effect on learning tasks	Parkhurst et al., 2013
		CX_3_CR1^CreER^:iDTR mouse line	>90	Fast repopulation, no negative outcome	Bruttger et al., 2015
	Pathology	PLX3397, CLs	70–90	Amelioration of tauopathy and neurotoxicity in AD mouse models	Asai et al., 2015
		PLX3397	93–97	Bigger infarct size and dysregulation of neuronal signaling leading to neuronal death in middle cerebral artery occlusion (MCAO)	Szalay et al., 2016
		CD11b-HSVTK mouse line	>90	Repression of inflammation in EAE	Heppner et al., 2005
		CD11b-HSVTK mouse line	90	No effects on ameloyd plaque formation or neuritic dystrophy in AD	Grathwohl et al., 2009
		CD11b-HSVTK^mt-30^ mouse line	50	No effect on disease outcome or neuronal survival in EAE	Gowing et al., 2008
		CD11b-HSVTK^mt-30^ mouse line	75	No effect on neuronal survival after mechanical injury	Gowing et al., 2006
		CD11b-HSVTK mouse line	80–90	No effect on seizure sensitivity in temporal lobe epilepsy (TLE)	Mirrione et al., 2010
		CD11b-HSVTK mouse line	75	Bigger infarct size and neuronal death in MCAO	Lalancette-Hebert et al., 2007


Another commonly identified microglia property is their repopulation capacity: around 1 week after depletion, the whole population is restored without obvious deficits in microglia or in other glial cell populations. With this experimental system, one group identified a local progenitor cell population in the brain parenchyma expressing the stem cell marker nestin that accounted for the rapid repopulation of microglia. The cellular characteristics of these progenitors were, however, not described further (Elmore et al., 2014, 2015).

Although demonstrating a similar microglia repopulation capacity, Bruttger et al. (2015) could not identify a separate repopulating progenitor pool but rather demonstrated a fast spreading distribution of local proliferating microglia that were able to escape the ablation treatment (∼5–10%). Some of these locally repopulating central nervous system (CNS) microglia also co-expressed nestin together with the microglial marker Iba1. This lead to the speculation that the nestin positive cells described in Elmore et al. (2014, 2015) are not a novel local progenitor pool but rather reactive microglia, as already shown for reactive astrocytes (Clarke et al., 1994). The observed identity differences in the repopulating pool could be due to distinct ablation approaches, as one analysis used PLX3397 to deplete microglia (Elmore et al., 2014, 2015), whilst the others used a mouse line in which iDTR-expression was driven by the fractalkine receptor CX_3_CR1 (CX3CR1^CreER^:iDTR) that is specifically expressed on microglia (Parkhurst et al., 2013; Bruttger et al., 2015). Myeloid cells in the periphery are equally targeted and depleted during PLX3397 treatment, which could, to some extent, affect the outcome of the analysis or even result in changes of the repopulating microglia cells. Another pitfall of the pharmacological approach could be CSF-1R expression on other cells within the brain that are not microglia which might therefore be targeted and ablated and hence lead to secondary effects in the brain. CX_3_CR1 is also highly expressed in peripheral macrophages (Geissmann et al., 2010). In the case of the inducible DTR-system, the peripheral cells were affected to a lesser extent. These cells have a high turnover from their stem cell source in comparison to resting microglia in the CNS and therefore lose the CreER-expression after tamoxifen treatment with time. Hence, DTA-application only affects persisting microglia but not the short-lived peripheral myeloid cells (Bruttger et al., 2015). The difference in the targeting specificity of these methods could account for the differences observed in these studies.

**FIGURE 1:**
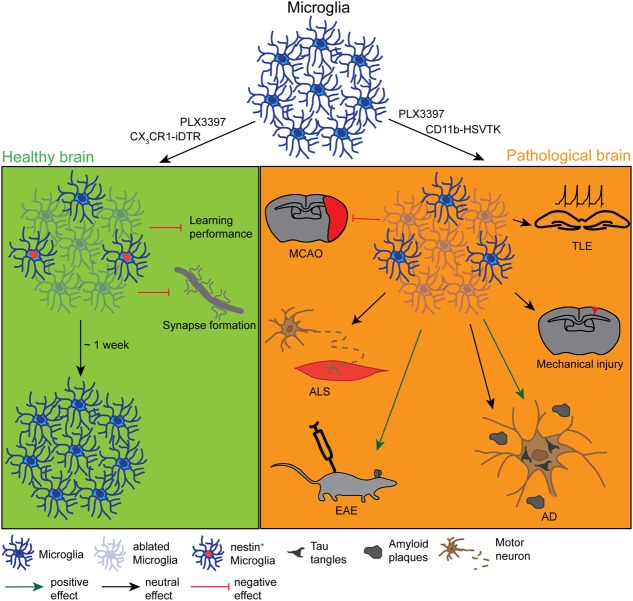
**Effects and dynamics of microglia ablation under healthy and pathological conditions.** Under physiological conditions (left green panel) successful microglia ablation has been achieved by the use of the pharmacological inhibitors PLX3397 and clodronate liposomes (CLs) or the CX_3_CR1-iDTR mouse lines. After approximately 1 week, the microglia population was completely restored from a nestin^+^ progenitor pool. Depending on the study, the ablation had either no physiological effect or it influenced the motor-dependent synapse formation and led to a poor performance in learning tasks. Under various pathological conditions (right orange panel) the ablation was also induced with PLX3397, CL or different CD11b-HSVTK mouse lines, however, with very controversial outcomes. While the microglia ablation had positive effects on the pathology of experimental autoimmune encephalomyelitis (EAE) or the tauopathy of Alzheimer’s disease (AD), it did not influence the pathology of amyloid plaque deposition in AD, mechanical injury, amyotrophic lateral sclerosis (ALS), or temporal lobe epilepsy (TLE) and even negatively affected the outcome of a middle cerebral artery occlusion (MCAO).

Although the aforementioned studies demonstrated comparable ablation and repopulation dynamics, the ideas about microglia function in the healthy adult brain are very different. Three studies performed in naïve, healthy young adult animals did not find any gross cellular changes or pathologies in microglia-ablated mice besides mild astrogliosis and a short cytokine storm where pro-inflammatory cytokines were highly up-regulated for a short time (Elmore et al., 2014, 2015; Bruttger et al., 2015). Parkhurst et al. (2013) on the other hand, reported a loss of motor-dependent synapse formation and decreased performance in learning tasks that seemed to be dependent on brain-derived neurotrophic factor (BDNF) signaling (**Figure 1**). Similarly, a loss of ∼80% of hippocampal microglia resulted in alterations in the social behavior of adult mice (Torres et al., 2016). These alterations were most likely induced by the direct microglia modulation of the number of functional neuronal synapses, as previously demonstrated by the same group using an *ex vivo* approach where microglia were depleted in slice cultures by addition of CLs, resulting in a direct increase in the synaptic activity of neurons (Ji et al., 2013). The major methodological differences between these approaches may explain the discrepancies in the results of these studies. These differences include the cloning strategy of the CX_3_CR1^CreER^- and the iDTR-locus and the induction protocol used for the tamoxifen-inducible ablations, which could both potentially result in a variable recombination rate with diverse readouts. An additional difference is the maintenance of CNS homeostasis after microglia ablation. While one study did not report disturbances in brain homeostasis (Parkhurst et al., 2013), the other observed mild astrogliosis and an increase in pro-inflammatory signals, which could also differently influence the synapse formation and behavior between the approaches.

In summary, the ablation studies of microglia in the adult healthy brain have revealed that these cells can, at least transiently, easily and efficiently be eliminated from the adult brain (summarized in **Table 1**), though surviving progenitors rapidly repopulate the depleted areas. Moreover, microglia homeostasis seems to play an important role in the ongoing synapse formation in the brain; these specific functions as well as mechanisms are however far from understood.

### Microglia Ablation under Pathological Conditions

Microglia are known to shift their phenotype from “resting” to an “activated” state upon any loss of brain homeostasis due to injury, infection, or neurodegenerative disease. This microglia activation is comprised of proliferation, migration toward a chemoattractant, reduction of cellular complexity as well as phagocytosis to clear the damaged tissue (Kreutzberg, 1996; Hanisch and Kettenmann, 2007; Kettenmann et al., 2011). The cellular response of microglia upon injury has been extensively described, however, both its functional impact on brain pathogenesis and its mechanism remain somewhat unclear. Because many contradictory findings have been reported with regard to microglia function after brain injury, microglia ablation studies continue to be performed (for summary and comparison, see **Table 1** bottom part).

In the same vein as microglia ablation studies under healthy conditions, the approaches used in disease models are mainly the well-established pharmacological (PLX3397 and CLs) as well as genetically-driven toxin methods, although predominantly the latter (see **Table 1** bottom part). Instead of the DTA- or DTR-system, pathological conditions have been investigated with a wildtype (TK) and a mutant (TK^mt-30^) thymidine kinase of the herpes simplex virus (HSV; HSVTK). The HSVTK is another commonly used suicide gene that only becomes toxic after the application of antiviral prodrugs like ganciclovir (GCV). These prodrugs are further processed into toxic triphosphates by cell-intrinsic kinases, resulting in the apoptotic death of cells. As TK mainly interferes with nucleosides and therefore disrupts DNA-replication, it specifically ablates proliferating cells rather than quiescent ones. As well as the wildtype thymidine TK, the mutant improved form of this enzyme (TK^mt-30^) exists. As an advantage, it shows an increased sensitivity to the applied drugs, allowing lower doses of the systemic drug and alleviating the male sterility that is a common problem observed in TK-transgenic mice (Fyfe et al., 1978; Black et al., 1996; Fischer et al., 2005). Unlike in the healthy CNS where microglia-targeting was achieved with the CX_3_CR1-promoter (see section Microglia Ablation under Healthy Conditions), all studies investigating the role of microglia in disease models use the CD11b-promoter that is also expressed in cells of myeloid origin, including both microglia and macrophages (Akiyama and McGeer, 1990; Dziennis et al., 1995). Most interestingly, in this system GCV treatment and the subsequent depletion of myeloid cells resulted in increased mortality (Grathwohl et al., 2009) that could only be circumvented by bone marrow transplantation. This method led to the advantageous situation that only microglia were depleted while keeping an intact blood circulation with wildtype myeloid cells.

Although microglia ablation was successful in the different studies, the quest for the role of microglia in disease models opened at least as many questions as it resolved. Asai et al. (2015) investigated the role of microglia in the tauopathy in Alzheimer’s disease (AD) using both a virus-mediated model for tau propagation and a transgenic model to ectopically express the human form of the tau protein. They addressed the role of microglia in AD by depleting these cells in adult mice (3.5 or 4.5 months). The depletion of 70–90% of microglia (depending on the region and the treatment) ameliorated the tauopathy as well as the neurotoxicity in these two mouse models of AD, indicating the importance of microglia in the progression of neurodegenerative diseases. Szalay et al. (2016) achieved an almost complete (93–97%) ablation of microglia. They addressed the functional outcome of microglial absence after Middle Cerebral Artery Occlusion (MCAO) in adult mice and found a striking increase in the infarcted area plus a dysregulated neuronal calcium response that led to neuronal death. This response was reversed with microglia repopulation, and therefore this study further emphasizes the importance of microglia in the pathological brain. While a >90% microglia ablation seemed to have a positive effect on clinical scores and inflammation in a model of experimental autoimmune encephalomyelitis (EAE, Heppner et al., 2005), it was neither beneficial nor deleterious in other disease models (see **Figure 1**). Although the tauopathy of AD could be resolved (as explained above), the 90% reduction of microglia did not affect amyloid plaque formation or neuritic dystrophy in the same disease model (Grathwohl et al., 2009), indicating that microglia only influence some pathological properties of the disease. Similarly, in a genetic model of amyotrophic lateral sclerosis (ALS, SOD1 mutation), a 50% reduction of the microglia population altered neither the disease outcome nor the degeneration of motor neurons (Gowing et al., 2008). Furthermore, toxin-induced cell death of about 75% of microglia after traumatic glossal nerve or cortical stab wound injury also did not affect neuronal survival (Gowing et al., 2006), indicating that a lack of any microglia ablation benefit to neuronal survival is not specified to a particular disease but in general to pathology. Although microglia are known to actively influence and modulate neuronal synapses under physiological conditions (Bessis et al., 2007), the absence of a substantial number of microglia (80–90% depending on the region) did not affect seizure sensitivity in a pilocarpine-induced model of temporal lobe epilepsy (TLE, Mirrione et al., 2010). Indeed, at least one study has shown the opposite: In addition to not changing the disease outcome, Lalancette-Hebert et al. (2007) reported a bigger infarct size and more damage to brain tissue after MCAO following an ablation of microglia of around 75%.

To explain these variable observations between ablation studies, the same aspects that complicate the readout of the approaches carried out under healthy conditions (see section Microglia Ablation under Healthy Conditions) certainly apply also here, and different disease models add another dimension of complexity. Although all these studies worked with CD11b-HSVTK transgenic mice, the ablation efficiency was highly variable, ranging from 50 to up to 90%, making a direct comparison between the models difficult. Besides the cloned mouse lines carrying different transgenes, the diverse injury paradigms that were performed in various CNS regions might play an additional role. The injury paradigms were performed at different ages of the experimental mice (ranging from 2 up to 4.5 months of age) and it could be possible that microglia function changes during the process of aging.

Although microglia are well known to react with similar morphological changes to different injuries and pathologies (Kreutzberg, 1996; Hanisch and Kettenmann, 2007; Kettenmann et al., 2011), it remains uncertain if the functional activation of microglia in response to different insults varies depending on the signals that are released from a specific type of pathology. The temporal aspect of the disease model might additionally influence the observations, depending on whether it is chronic (like in the case of AD and ALS) or acute (e.g., MCAO and mechanical brain injury). The idea that subtypes of activated microglia affect disease outcome in different directions has long been proposed. Classically-activated M1-like microglia were long thought to have mainly pro-inflammatory functions and were therefore considered to be “bad” for the outcome of the pathology, while the alternatively-activated “good” M2-like microglia were considered to be important for the anti-inflammatory functions that minimize pathology (Kigerl et al., 2009; Varnum and Ikezu, 2012; Miron et al., 2013; Zhou et al., 2014). New research has called this concept of clearly-polarized classes of microglia into question, as the activation state of microglia might be dependent on the stimulus (Kim et al., 2016; Ransohoff, 2016). Transferring these findings to the above described ablation studies, it is still not known whether the TK-induced ablation is preferentially targeting a polarized subset of cells or the whole microglial population, which would differentially influence the outcome of the disease. Furthermore, it is unclear whether differentially-activated macrophages become two different populations that co-exist during the whole course of pathology or if they can inter-convert at different stages (Varnum and Ikezu, 2012; Weisser et al., 2013). If the latter hypothesis holds true, the timing of the onset of the microglia ablation studies would play another important factor that would need further consideration.

Taken together, it can be concluded that using different approaches, activated microglia can also efficiently be depleted in various kinds of brain pathologies, but the specific roles of these cells in disease must be clarified with further studies.

## Astrocytes

### Astrocyte Ablation under Healthy Conditions

Astrocytes represent the most abundant fraction of glial cell types in the adult brain (Kettenmann and Ransom, 2005). Amongst all glial cell types, several of the functional roles of astrocytes in the healthy adult brain are already well described. These functions are broad, spanning many aspects of brain physiology and are so numerous that addressing all of them is far beyond the scope of this review. In short, the maintenance of water and ion homeostasis, the participation in the tripartite synapse as well as the contribution to the BBB maintenance are among the most important astrocytic functions (Kimelberg, 2010; Kimelberg and Nedergaard, 2010).

Astrocytes are, assumedly due to their high abundance, the best studied and most well described glial cell population in the adult CNS. Therefore the idea to specifically ablate astrocytes in order to find out their specific functions has been developed much earlier compared to other glial cell populations. Nevertheless, there is only a small amount of ablation studies in the field that target the astrocyte population under physiological conditions.

To specifically deplete the astrocyte population in the adult healthy brain, again both pharmacological as well as genetic approaches are available and have been used (see **Table 2**). The glutamate homologue L-α-aminoadipate (L-AAA) is the only pharmacological approach to ablate astrocytes so far and was shown to be a specific toxin for astrocytes rather than for neurons or other glial cell types when locally injected into the site of interest (Takada and Hattori, 1986; Nishimura et al., 2000).

**Table 2:** Astrocyte ablation approaches under healthy and pathological conditions.

Cell type	Condition	Method	Efficiency	Physiological effect	Reference
Astrocytes	Health	L-AAA	100% at injection site	No effect on neuronal density, some microglia reactivity	Khurgel et al., 1996
		GFAP-NTR mouse line	Nearly all in the cerebellum	Neuronal degeneration of granular cells in the cerebellum as Bergmann glia were the major targets and motor discoordination	Cui et al., 2001
		GFAPCre:iDTR mouse line and GFAPCreERT2-DTA mouse line	73% in the spinal cord	Axonal degeneration and limb paralysis due to increase in ROS/NOS	Schreiner et al., 2015
	Pathology	GFAP-HSVTK mouse line	Not directly specified	Failure of scar formation and increased immune invasion after EAE	Voskuhl et al., 2009
		GFAP-HSVTK mouse line	>95% in lesion area	Increased inflammation and higher damage after mechanical SCI	Faulkner et al., 2004
		GFAP-HSVTK mouse line	Not directly specified	Increase in injury size and inflammation after mechanical cortical injury	Bush et al., 1999; Myer et al., 2006
		GFAP-HSVTK mouse line	Not directly specified	Less immune cell activation and worsening of myelin debris clearance after cuprizone-induced demyelination	Skripuletz et al., 2013
		GFAP-HSVTK mouse line	Not directly specified	No effect on neuronal survival, disease duration and outcome in neurodegeneration	Lepore et al., 2008


Suicide gene expression exploited for this cell population has mainly made use of the intermediate filament glial fibrillary acidic protein (GFAP) promoter that is only expressed by a subset of astrocytes in specific regions of the healthy brain (Takamiya et al., 1988; Cahoy et al., 2008). Either a constitutively active or tamoxifen-inducible GFAP promoter was used in combination with the already mentioned DTA-system (GFAPCreERT2-DTA mouse line, Schreiner et al., 2015) or with the bacterial nitroreductase (NTR, GFAP-NTR mouse line). NTR is another suicide gene where expression alone is not toxic, but the enzyme metabolizes systemically given prodrugs into a toxic agent. The advantage of the NTR over the suicide genes mentioned earlier is that they are not only targeted to proliferating cells, producing toxic agents that are independent of proliferation (Knox et al., 1993).

Khurgel et al. (1996) used local ablation of astrocytes to reveal their role in the healthy amygdala of adult rats. Injections of L-AAA successfully generated a 100% astrocyte-deprived zone with a size of 200–500 μm within 45 h after injection which remained for further 7 days. The depletion was accompanied by some microglial reactivity but without effects in neuronal density.

In contrast to the functional outcome after the drug-induced astrocyte ablation (Khurgel et al., 1996), genetic ablation resulted in severe neuronal degeneration (Cui et al., 2001). With this specific approach, predominantly Bergmann glia, an astrocytic subtype located in the cerebellum, were targeted in mice. The gross ablation of Bergmann glia resulted in severe developmental problems in the motor coordination of these mice, resembling a hallmark of cerebellar dysfunction. The regional specificity of the ablation observed might be explained by how the mouse line they generated was used, as NTR-expression was detected mainly in these cells. Recently, similar findings were obtained in the spinal cord using the tamoxifen inducible human GFAP-driven diphtheria toxin A (DTA)-expression (GFAPCreERT2-DTA), where local ablation of 73% of GFAP-expressing astrocytes was achieved in the cervical spinal cord in young adult mice (Schreiner et al., 2015). Shortly after tamoxifen application, mice suffered axonal degeneration accompanied by paralysis of all limbs, while the BBB remained intact. The mechanism of action was ascribed to the failure to buffer reactive oxygen and nitrogen species (ROS/NOS), as the mice lack astrocytes that usually clear the brain from these damaging substances. These results raise the hypothesis that a GFAP-expressing subset of astrocytes is crucial for the maintenance of neuronal health and integrity, while other astrocytes are devoted to other functions, such as the BBB maintenance. In contrast, another study using the non-inducible DTA-system with an ubiquitous astrocyte specific promoter other than GFAP, namely the aldehyde dehydrogenase 1 family member L1 promoter (Aldh1L1), depleted ∼30% of all astrocytes in the spinal cord and did not describe any deficits in the neuronal support (Tsai et al., 2012). The differences between these two studies might lie in the huge variation in ablation efficiency (73% vs. 30%). In addition, the latter study was carried out much earlier during development at embryonic stages. This could add to the different outcomes, as these early appearing astrocytes might have other functions in the CNS than during adult stages, or because the compensation mechanisms during development are more supportive than in the adult.

Particularly for astrocytes, the use of different ablation models (GFAPCreERT2-DTA vs. Aldh1L1-Cre-DTA vs. GFAP- NTR vs. L-AAA), different areas of interest (spinal cord vs. cerebellum vs. amygdala) and different ages (development vs. adult) are hampering the formation of a consistent concluding statement for these studies. Astrocytes exhibit many different morphologies and accordingly different functions depending on the CNS region (Robel et al., 2011). Using the GFAP-promoter as a driving-force of astrocyte ablation is biased as GFAP is not uniformly expressed in all astrocytes in the CNS, e.g., astrocytes located in the cerebral white matter but not the gray matter express GFAP (Cahoy et al., 2008; Robel et al., 2011). Therefore genes expressed under the GFAP-promoter might only be displayed in a subset of astrocytes with potentially specific functions that are different from other resident astrocytes. Moreover, these studies used different readouts in their analysis and some aspects, like the BBB closure or neuronal damage, might simply have been overlooked. Thus far, no study using genetic GFAP-driven approaches has achieved ablation of cortical astrocytes under physiological conditions in the adult brain, most likely due to the lack of promoter-expression in these cells. To achieve ablation of cortical astrocytes, other promoters that are (strongly) expressed in astrocytic populations of the cerebral cortex should be considered.

**FIGURE 2:**
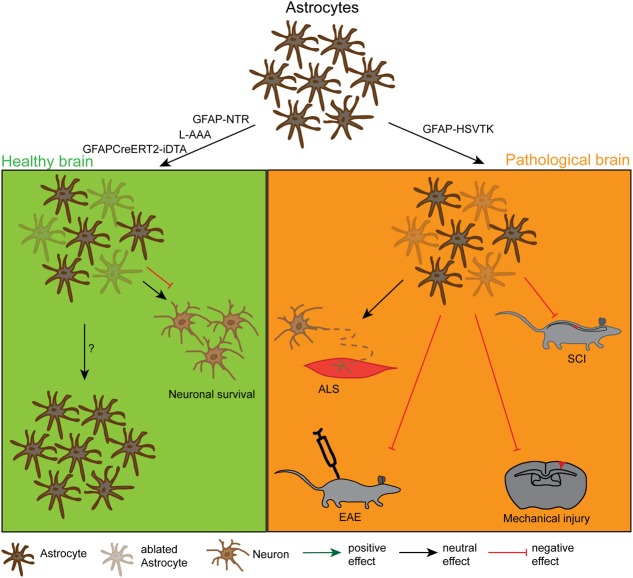
**Effects and dynamics of astrocyte ablation under healthy and pathological conditions.** Under physiological conditions (left green panel) successful astrocyte ablation has been achieved by the use of the pharmacological drug L-α-aminoadipic acid (L-AAA) or the GFAPCreERT2-DTA and the GFAP-NTR mouse lines. For the astrocytes, the repopulation kinetics have not been analysed in detail yet, but these cells were also shown to repopulate the depleted area. As a functional outcome, the ablation did either not show an effect or had a negative effect on neuronal survival in the cerebellum and the spinal cord. Under pathological conditions (right orange panel) astrocyte ablation was solely achieved by the use of the GFAP-HSVTK mouse line. However, with this model only the pool of proliferating astrocytes can be depleted. The reduction of scar forming astrocytes in general had a negative outcome for the injury size and severity in spinal cord injury (SCI), mechanical brain injury and EAE. It did not affect the pathology in a model of ALS, but this could be due to the low amount of proliferating astrocytes in this model.

In summary, these studies demonstrated different methods to induce the depletion of astrocytes from at least some parts of the CNS and support the idea of astrocytes being important for neuronal and axonal integrity on a functional basis (**Figure 2**; **Table 2**). These ablation models could be exploited in further experiments to address specific interaction points between astrocytes and neurons. Most interestingly – in contrast to other glial cell types – no studies exist that deal with the repopulation dynamics of astrocytes after ablation or the long term effects of this ablation, which could also be a very illuminating topic of interest.

### Astrocyte Ablation under Pathological Conditions

Upon injury, astrocytes undergo a series of morphological and functional changes that are commonly summarized by the term “astrogliosis”. Astrogliosis displays a very broad spectrum of reactivity, the hallmarks thereof being the upregulation of structural proteins like GFAP or vimentin and the hypertrophy of both the cell body and the processes (Wilhelmsson et al., 2004; Sofroniew and Vinters, 2010). Additionally, some quiescent astrocytes re-enter the cell cycle (Buffo et al., 2005), although this occurs much later and to a lesser extent than in microglia or NG2-glia. Reactive astrocytes are also known to elongate around the lesion core (Wanner et al., 2013) and to release a cascade of inflammatory signals that can strongly affect the pathological outcome (Sofroniew, 2015). Interestingly, as shown by repetitive *in vivo* imaging, astrocytes are – unlike microglia and NG2-glia – unable to migrate into the injury core after mechanical injury (Bardehle et al., 2013). Although many aspects of astrocyte-specific reactions to injury are already known, their precise role in scar formation under different pathological conditions remains unresolved. As this question has long been of great interest, studies that specifically ablate these scar forming astrocytes have already been reported almost 20 years ago.

Notably, in contrast to physiological conditions, no studies have attempted to ablate astrocytes under pathological conditions using pharmacological approaches, in contrast to genetic ones that were normally used (see **Table 2** bottom part). The population targeted when utilizing the GFAP-promoter in pathological conditions is predicted to be much higher and might even go beyond the specific subset of cells observed in healthy tissue. This is because a huge proportion of cortical astrocytes upregulate GFAP upon any changes in brain homeostasis (Takamiya et al., 1988). Using the murine GFAP-promoter to target specific cells, many studies also use the HSVTK suicide gene (GFAP-HSVTK mouse line; originally created by Bush et al., 1998) as an inducer of apoptosis and to analyze the role of astrocytic scar formation. The pathological conditions examined with this approach spanned a broad range from neurodegenerative diseases to traumatic brain injury.

With this ablation model, already within 1 week a myelin oligodendrocyte glycoprotein (MOG)-induced model of EAE, which mainly shows pathology in the spinal cord, showed robust astrocytic death that resulted in a failure of tissue scar formation, increased immune cell infiltration as well as a worsened clinical score (Voskuhl et al., 2009). Similarly, in the spinal cord, >95% astrocyte ablation that was achieved within 7 days after small stab wound and crush injuries led to increased tissue damage with higher cell death and increased inflammation (Faulkner et al., 2004). Mechanical injury to the brain identified similar findings to studies in the spinal cord, but with greater effect. The brain suffered more detrimental injury and greater damage, which was induced by robust astrocyte ablation after 7 days of ganciclovir treatment (Bush et al., 1999; Myer et al., 2006).

All these studies – independent of the pathological conditions – presented an exacerbation of the disease outcome after the ablation of astrocytes, with only two exceptions presenting contradictory results. Skripuletz et al. (2013) analyzed the outcome of the astrocyte ablation in a cuprizone-induced demyelination model. In contrast to the other studies, less immune cell activation – in this case specifically microglia – could be observed, resulting in less efficient myelin clearance. One explanation for this different outcome could be due to the cell types involved: while all other pathological models include the recruitment of peripheral immune cells for debris clearance, the cuprizone model mainly relies on resident brain cells (Kipp et al., 2009). Moreover, astrocytes might play different roles in diverse pathological conditions. Along the same line, no effect could be found on disease onset, duration, neuronal loss or motor function in astrocyte ablated mice for two different models of neurodegenerative diseases (injection of the neuroadapted Sindbis virus (NSV) inducing neuronal death as well as in a genetic model of ALS) (Lepore et al., 2008). However, the number of proliferating astrocytes in these neurodegenerative disease models is relatively low in comparison to the other models of pathology. These contradictory results might simply be an effect of low numbers of ablated astrocytes and more remaining ones that are able to react, rather than of their function.

In summary, the ablation of astrocytes under pathological conditions has given a clear functional readout for the first time: astrocytes play a crucial role in the maintenance of the diseased tissue and in the inhibition of secondary tissue damage by blocking inflammation (**Figure 2**; **Table 2**). This interpretation has to be taken with care, as only proliferating astrocytes were ablated in all the approaches described above, while the non-proliferating cells retained normal function that might be different or even opposing to that of the proliferating ones. Moreover, it is now textbook knowledge that although scar forming astrocytes are beneficial to injury, they also have detrimental effects on tissue regeneration (Pekny and Pekna, 2014; Sofroniew, 2015). These aspects were only slightly addressed in the experiments mentioned above, giving space for further ablation approaches in the future.

## Oligodendrocyte Lineage

### NG2-Glia/Oligodendrocyte Ablation under Healthy Conditions

Although NG2-glia are precursors of mature oligodendrocytes within the oligodendrocyte lineage, they are often considered an independent glial population due to their additional characteristics. In relation to the long history of neuroscience, NG2-glia are a relatively young cell type in terms of discovery with identification only 30 years ago (ffrench-Constant and Raff, 1986). Although a great amount of work has been performed on these cells, the cellular function of NG2-glia – at least in the adult brain – remains largely a mystery. However, many cellular characteristics have been described: NG2-glia are part of the oligodendrocyte lineage and keep generating mature myelinating oligodendrocytes throughout lifetime (Dimou et al., 2008; Rivers et al., 2008; Psachoulia et al., 2009; Simon et al., 2011). Most interestingly NG2-glia in the adult rodent brain form a tight homeostatic network in which the cell numbers are maintained under physiological conditions. As soon as one cell has been lost due to either differentiation or cell death, the remaining gap is immediately replaced by a neighboring cell (Hughes et al., 2013). Furthermore, NG2-glia are the only highly proliferative cells in the brain parenchyma (Dimou et al., 2008), giving them characteristics analogous to stem cells. The most curious aspect of NG2-glia is their ability to form functional synapses with neurons, originally discovered in the hippocampus (Bergles et al., 2000) but now also described in other parts of the brain (Karadottir et al., 2005; reviewed in Sun and Dietrich, 2013). The function of these synapses is not well understood. One interesting aspect of these synapses is the directionality, as NG2-glia are only able to receive neuronal signals but cannot create action potentials on their own and propagate them further (De Biase et al., 2010). The ablation approaches that are discussed in this section give some insight in the role(s) of NG2-glia in the adult brain.

Further on in the lineage, the function of mature oligodendrocytes is clear: they are the myelin-producing cells that insulate axons to allow a rapid saltatory conduction and give trophic support to axons (reviewed in Nave, 2010). But as high numbers of presumably non-myelinating oligodendrocytes are also present in sparsely myelinated brain regions like the gray matter of the cerebral cortex, some functional aspects may have been overlooked. To address this question, specific oligodendrocyte ablation approaches may deepen our understanding of the functions of these cells.

**FIGURE 3:**
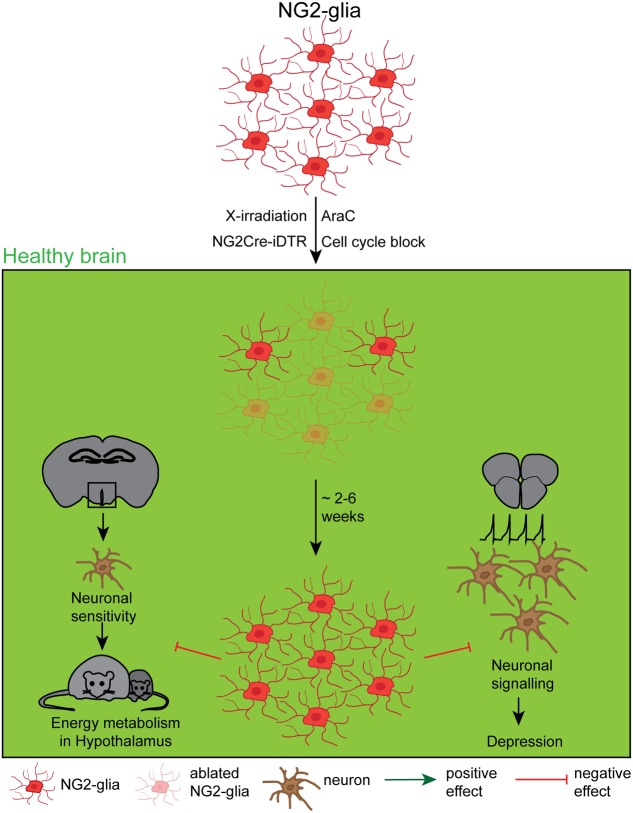
**Effects and dynamics of NG2-glia ablation under healthy conditions.** Although a relatively unknown cell type, several approaches to deplete NG2-glia under physiological conditions, including the mitotic blocker arabinofuranosyl cytidine (AraC), a genetic cell cycle block, X-irradiation and the NG2Cre/iDTR mouse line. The very efficient repopulation occurred either immediately or between 2 and 6 weeks after the ablation, depending on the study. Although the function of NG2-glia has long been a mystery, recent studies showed that the NG2-glia ablation negatively affects the leptin-dependent energy metabolism and leads to a depression like behavior.

Although NG2-glia were only discovered a short time ago (ffrench-Constant and Raff, 1986), several approaches to deplete these cells from the adult brain have already been developed. As NG2-glia are well characterized on the cellular level, these approaches mainly aimed to clarify the physiological function of these cells. However, most of these approaches have been rather disappointing until recently, both in terms of achieving a NG2-glia-free brain as well as in identifying the physiological function of these cells. The ablation of NG2-glia proved to be more difficult in comparison with other glial cell populations: due to their tightly regulated homeostasis (Hughes et al., 2013) the cells escaping ablation immediately react, increasing their proliferation rate to replace the lost cells. So far no method has successfully ablated all NG2-glia over a long period of time, as can be achieved successfully for microglia (compare **Table 3** and **Figure 3**). Although technically quite different, all of the ablation approaches for NG2-glia commonly demonstrated the highly efficient repopulation capacity of resident NG2-glia, ranging from two to 6 weeks depending on the study. Two-photon *in vivo* imaging demonstrated the mechanism by which these cells become aware of their need for proliferation: they are able to sense cell-free gaps with their fine filopodia, triggering their migration or proliferation in order to fill the gap (Hughes et al., 2013). Interestingly, these repopulating cells seem to be less branched and occupy a smaller surface area (Birey and Aguirre, 2015). However, this could change over time as the cells re-grow and re-gain their original cellular complexity.

**Table 3:** NG2-glia ablation and differentiation block approaches under healthy conditions.

Cell type	Condition	Method	Efficiency	Physiological effect	Reference
NG2-glia	Health	X-irradiation	60–90%	Fast and efficient repopulation, no further effects analyzed	Chari and Blakemore, 2002; Irvine and Blakemore, 2007
		AraC infusion	Almost 100% in affected region	Fast and efficient repopulation, no further effects analyzed	Robins et al., 2013
		NG2Cre/iDTR mouse line	80%	Fast repopulation, decrease in FGF2 signaling and induction of depression like behavior	Birey et al., 2015; Birey and Aguirre, 2015
		AraC infusion, X-irradiation, genetic depletion model	50–100%	Negative effect on leptin-sensitive neurons and energy metabolism	Djogo et al., 2016
NG2-glia oligodendrocyte differentiation	Health	Genetic depletion model	∼90%	Elongation of nodes of Ranvier, deceleration of conduction velocity and motor dysfunctions	Schneider et al., 2016
		Genetic depletion model	100%	Impairment of early complex motor skill learning	McKenzie et al., 2014; Xiao et al., 2016


Although the repopulation capacity of NG2-glia – independent of the ablation approach – has fundamentally proven to be fast, it declines with aging (Chari et al., 2003; Birey and Aguirre, 2015). In correlation with their slowing physiological cycling behavior, the cell cycle length increases with aging (Psachoulia et al., 2009; Young et al., 2013). Early studies depleting NG2-glia both in the spinal cord as well as in the brain with high-dose X-irradiation demonstrated an efficient repopulation within four to 6 weeks following the ablation treatment (Chari and Blakemore, 2002; Irvine and Blakemore, 2007). The magnitude of the cell death of NG2-glia as well as their repopulation capacity were, however, more efficient in developmentally younger areas like the telencephalon (90% of ablation efficiency) compared with older ones like the diencephalon (60% of ablation efficiency). The nature of these regional differences might either lie in a different cell cycle length of NG2-glia, as X-irradiation mainly affects fast cycling cells, or it may be an issue of different developmental origins or regional heterogeneity of those cells (Kessaris et al., 2006; Viganò et al., 2013). The use of a single cell type-specific antibody to estimate the efficiency of depletion can lead to an overestimation since some proteins might be down-regulated upon a change in brain homeostasis, as shown for neurons after injury (Pignataro et al., 2008; Jaenisch et al., 2010). If this also holds true for NG2-glia, the X-irradiation method would solely lead to a failure to detect these cells with the use of an anti-NG2 antibody although they are still present. NG2-glia are known to be the only proliferating cells outside the neurogenic niches in the healthy adult brain and therefore suspected to be the only cell type responding to X-irradiation. However, after a mild irradiation injury, other cells like microglia and astrocytes become reactive upon any cellular death, potentially triggering their proliferation in response to damage. This would make them sensitive to irradiation-induced cellular death, hence weakening the cell type specificity of this method.

A more specific and therefore more elegant way to follow the dynamics of ablation and repopulation of NG2-glia *in vivo* by overcoming the pitfalls of detection with different antibodies, is the use of genetically modified mouse lines to intrinsically label NG2-glia. In a recent study, a NG2-CreER:tdTomato mouse line permanently expressing the red fluorescent marker “tomato” under the control of the NG2-promoter after tamoxifen induction was used (Robins et al., 2013). As this tamoxifen-inducible marker is permanently expressed under the ubiquitous Rosa26-promoter, the downregulation of this locus is very unlikely to happen. Combining this mouse line with the intraventricular administration of AraC, a toxic agent interfering with the cellular DNA synthesis and inducing cell death in fast cycling but not slow or non-cycling cells (Doetsch et al., 1999), led to rapid ablation of almost 100% of reporter-positive NG2-glia in the hypothalamus. This ablation was then followed by a subsequent, complete repopulation within 2 weeks. Furthermore, with the use of BrdU-labeling experiments, insights were provided that the repopulation of NG2-glia exclusively occurs through proliferation of surviving adjacent NG2-glia located in an area without AraC diffusion, but not from a NG2-negative stem cell source.

These first ablation studies fundamentally characterized the repopulation capacity of NG2-glia, but did not answer the question about the NG2-glia function in the adult brain. Two recently published NG2-glia ablation studies have so far directly addressed the functional outcome of an at least transient lack of NG2-glia (Birey et al., 2015; Djogo et al., 2016, see also **Figure 3**). Birey et al. (2015) reported that NG2-glia secrete the fibroblast growth factor 2 (FGF2) that directly influences the neuronal glutamatergic transmission and the astrocytic glutamate uptake in the prefrontal cortex. Ectopic DT application in NG2Cre/iDTR mice expressing the DTR under an ubiquitous promoter whose expression is driven by the NG2-promoter, induced a selective ablation of 80% of NG2-glia in the prefrontal cortex and resulted in a reduced FGF2 cell-to cell signaling what induced a depression-like behavior in the ablated mice. This work therefore indicates that NG2-glia have a direct influence on the functionality and the properties of the neuronal network. The mechanisms of this interaction still remains, however, a speculation. Djogo et al. (2016) used different methods to successfully ablate NG2-glia in the hypothalamus (ranging from 50% in general to 100% in the treated area): (1) AraC infusion into the 3rd ventricle, (2) genetically induced apoptosis that is driven by the lack of a necessary cell cycle protein Esco2 in NG2-glia, as well as (3) X-irradiation. In this work, it could be demonstrated that under physiological conditions NG2-glia in the hypothalamus contact dendritic processes of leptin receptor neurons which degenerate upon NG2-glia ablation, reducing leptin signaling. Hence, NG2-glia are essential to maintain the function of leptin receptor neurons in the hypothalamus, therefore proving for the first time a direct role for NG2-glia in the maintenance of the thalamic energy metabolism.

NG2-glia are part of the oligodendrocyte lineage and are continuously differentiating into mature/myelinating oligodendrocytes also throughout adulthood; hence the fate of these two cell populations is highly connected (Dimou et al., 2008; Simon et al., 2011; Young et al., 2013). The need for these newly generated oligodendrocytes in the adulthood remains, however, not well understood. A recent study tested the hypothesis whether chronic NG2-glia ablation also influences the oligodendrocyte differentiation and assessed potential functional consequences (Schneider et al., 2016). This study took advantage of the above mentioned genetic ablation model in which the deletion of the cell cycle protein Esco2 driven by the Sox10-promoter induces apoptosis of proliferating NG2-glia. This approach achieved a chronic diminishment of 90% of recombined NG2-glia over a period of 16 weeks in the white matter of the cerebral cortex while the total number of NG2-glia remained stable. This depletion of recombined cells yielded a reduced oligodendrogenesis that further resulted in an elongation of the nodes of Ranvier, reduction of the saltatory nerve conduction as well as in motor dysfunctions, therefore demonstrating the importance of constant oligodendrogenesis in the adult brain. Another study did not directly ablate oligodendrocyte lineage cells, but also blocked this lineage progression by knocking out the transcription factor Myrf under the platelet derived growth factor receptor alpha (PDGFRα)-promoter, resulting in the failure of NG2-glia to differentiate to almost 100% (McKenzie et al., 2014; Xiao et al., 2016). In line with the above mentioned study, it could be demonstrated that the lifelong oligodendrogenesis is required for physiological function of the brain – in this case in the very early stages of complex motor skill learning.

**FIGURE 4:**
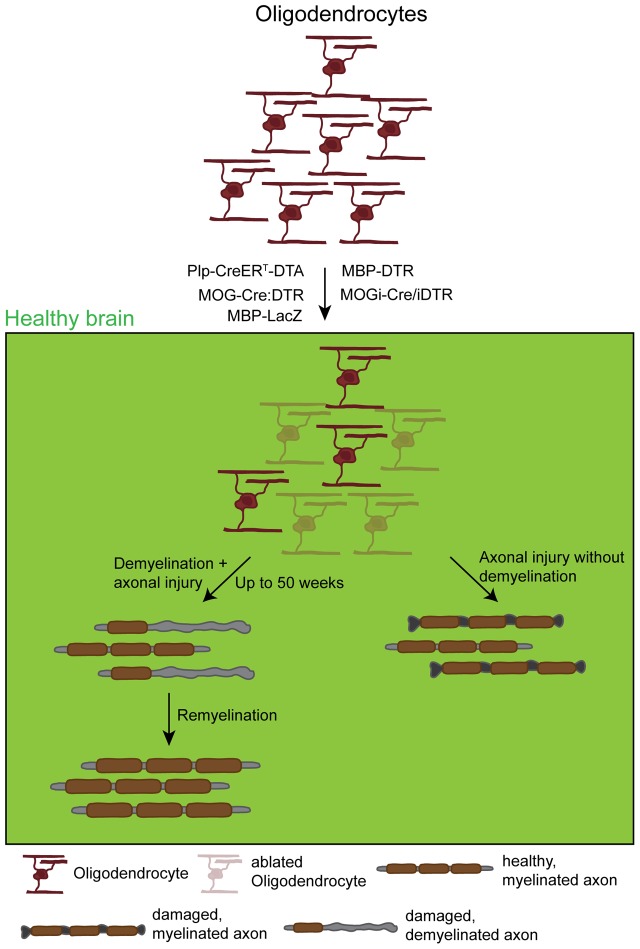
**Effects and dynamics of oligodendrocyte ablation under healthy conditions.** Under physiological conditions successful oligodendrocyte ablation has been achieved by the use of several approaches: the PLP-CreER^T^-DTA, the MOG-Cre:DTR, the MBP-DTR, the MBP-LacZ, and the MOGi-Cre/iDTR mouse lines. Very commonly, although very diverse in the use of promoters and suicide genes, these approaches induced oligodendrocyte death that in most cases resulted in primary demyelination followed by secondary induced neuronal damage. These observations were in most cases accompanied by a behavioral phenotype resulting from demyelination. This phenotype could, however, take up to 50 weeks to appear, depending on the model. After a longer time, demyelination was followed by a spontaneous remyelination. Only one study observed axonal damage without global demyelination that could be due to the loss of trophic axonal support.

While the functional outcome of a NG2-glia ablation is just at the beginning of being understood, the role of oligodendrocytes and their ablation in the adult brain has been subjected to studies for several years (compare **Table 4** and **Figure 4**), not only in the rodent CNS (Vanderluit et al., 2000; Ghosh et al., 2011; Locatelli et al., 2012; Oluich et al., 2012; Gritsch et al., 2014; Traka et al., 2016) but also in other vertebrates like, e.g., in zebrafish (Chung et al., 2013) and in xenopus (Kaya et al., 2012). Furthermore, a broad variety of demyelination models that are used to study Multiple Sclerosis (MS) like, e.g., cuprizone is also to a great extent killing mature oligodendrocytes (Praet et al., 2014), but as their primary purpose is not the study of the cell ablation, they are not further discussed in this review.

**Table 4:** Oligodendrocyte ablation approaches under healthy conditions.

Cell type	Condition	Method	Efficiency (%)	Physiological effect	Reference
Oligodendrocytes	Health	Plp-CreER^T^-DTA mouse line	∼50	Demyelination, remyelination but secondary axonal damage with motor impairment and seizures	Traka et al., 2016
		MOGi-Cre/iDTR mouse line	∼60	Demyelination, gait disturbances, and tremor	Ghosh et al., 2011
		MOG-Cre;DTR mouse line	∼80	Neuropathic pain and global demyelination independent of immune system	Gritsch et al., 2014
		Mbp-DTR mouse line	26	Axonal damage without demyelination	Oluich et al., 2012
		Mbp-LacZ mouse line	∼50	Demyelination, remyelination, no functional outcome analyzed	Vanderluit et al., 2000
		MOGi-Cre/iDTR mouse line	60	Demyelination, motor dysfunctions, no autoimmunity	Locatelli et al., 2012


Since oligodendrocytes do not have particular unique features like being proliferative, all of the used approaches are taking advantage of a toxin-induced ablation system amongst which the DTA-system is the most common one in combination with promoters that are specific for oligodendrocytes (summarized in **Table 4**). DTA toxicity in oligodendrocytes was, e.g., directly activated by tamoxifen-dependent Cre-induced recombination under the proteolipid protein (Plp)-promoter (Plp-CreER^T^-DTA mice), allowing a long-lasting reduction of oligodendrocytes by 50% in the brain stem that could even be detected at >50 weeks after recombination. This was accompanied with the development of an autoimmune response against myelin (Traka et al., 2016). A much faster oligodendrocyte death could be achieved by the expression of the diphtheria-toxin receptor (DTR) under the MOG-promoter, where an ectopic DTA application for 24 days resulted in 60% oligodendrocytic death in the corpus callosum subsequently leading to axonal damage (Ghosh et al., 2011). Using a similar system with the DTR-expression under the same promoter, another study could even achieve an oligodendrocyte ablation efficiency of around 80% that was accompanied with neuropathic pain (Gritsch et al., 2014). A similarly efficient approach with a tamoxifen inducible MOGi-cre:iDTR mouse line could also achieve a stable oligodendrocyte ablation of around 60% throughout the brain (Locatelli et al., 2012). Furthermore, in another model for the effective ablation of oligodendrocytes the DTR expression under the myelin basic protein (MBP)-promoter was used, resulting in an oligodendrocyte reduction of around 30% throughout the CNS (Oluich et al., 2012). Already some time ago, Vanderluit et al. (2000) developed a very rare, but unique system in which oligodendrocytes express the suicide gene ß-galactosidase (LacZ) under the MBP-promoter. Local application of fluorescein-di-ß-galactopyranoside/3-amino-9-ethyl-carbazole (FDG/AEC) forms a toxic precipitate after illumination that induces the death of 50% of oligodendrocytes at the injection site in the spinal cord. This LacZ-system approach allows the focal ablation of cells that can even be controlled in size, while all the other described studies used systemic drug application and hence targeted and ablated oligodendrocytes in the complete CNS. However, this study did not report any functional outcome of the ablation but might be a good tool for future experiments.

Despite the differences in the ablation approach and possibly the functional readout, all studies have in common that the death of myelinating oligodendrocytes leads to a primary demyelination with a persisting secondary induced axonal damage and a subsequent spontaneous remyelination (**Figure 4**). In most of the studies the demyelination and the axonal damage are accompanied by severe motor dysfunctions like tremor, ataxia as well as weight loss or even the development of seizures. Although there is a severe loss of myelin and an accumulation of myelin debris, one study reported about the absence of the development of an autoimmune-response, what is generally thought to happen during MS pathology (Locatelli et al., 2012). Due to the widespread myelin loss, the biology of these models all together creates a perfect tool for studying remyelination besides analyzing the outcome of oligodendrocyte death. Interestingly, Oluich et al. (2012) reported the loss of oligodendrocytes with axonal damage but without a severe and widespread demyelination, supporting the idea of a further function of oligodendrocytes providing a trophic support to axons. A possible reason for the different outcomes might be the recombination efficiency: while the ablation rate reached between 50 and 80% in most studies, the latter one ablated less than 30% of the oligodendrocytes that might not be enough to result in a global demyelination but already induce some axonal damage.

In summary, the specific ablation of both NG2-glia as well as oligodendrocytes using different approaches proved to be similarly effective than for the other glial cell types; in case of the NG2-glia at least transiently as they repopulate very fast (see **Table 3**). While for the oligodendrocytes the functional outcome of myelin loss and axonal damage seemed to be quite foreseeable (**Table 4**), the role of NG2-glia in the adult brain remains open and further ablation studies could give a deeper insight.

### NG2-Glia/Oligodendrocyte Ablation under Pathological Conditions

Many cellular characteristics of NG2-glia and their activation upon different pathologies have been well described. Like astrocytes and microglia, NG2-glia were shown to react to various kinds of pathological insults, however, only when accompanied with an opening of the BBB (Rhodes et al., 2006; Wang and He, 2009; Sirko et al., 2013; Dimou and Götz, 2014). Two-photon *in vivo* imaging studies even revealed a very heterogeneous reaction of NG2-glia to injury: cell migration, proliferation, hypertrophy, and even combined reactions are possible (Hughes et al., 2013, von Streitberg and Dimou, unpublished observations). Moreover, it became clear that NG2-glia strongly increase in number and align around the lesion site as part of the glial scar (Levine et al., 2001; Simon et al., 2011). Besides these observations, the cellular role of NG2-glia under pathological conditions remains unresolved – comparable to the healthy brain.

Interestingly, unlike the other glial cell types, so far there are no published studies that specifically ablate NG2-glia under pathological conditions. However, as the ablation approaches under healthy conditions are quite successful – at least transiently – they seem to be a very promising tool to further tackle the functional role of these cells in pathology.

In a similar way, there are also no studies where mature and myelinating oligodendrocytes have been specifically depleted from the adult brain under pathological conditions. Probably these experiments are also very unlikely to be performed in the future, as oligodendrocytes were not shown to react to different kinds of injury besides providing new myelin during tissue repair, but remain rather stable. Furthermore, the death of oligodendrocytes induces global demyelination as well as axonal defects already under healthy conditions (Vanderluit et al., 2000; Ghosh et al., 2011; Gritsch et al., 2014; Traka et al., 2016), which would in the case of pathology always result in a worse outcome and hence not be beneficial.

Summarizing this section, nothing has been published regarding ablation studies of oligodendrocyte lineage cells under pathological conditions. However, as especially for NG2-glia the already established methods have proven to be effective, very promising studies during pathological conditions will likely be investigated in the future.

## Discussion and Outlook

This review summarizes the tremendous work of the last decades on the various ablation approaches in all types of glial cells in the adult brain (see also **Tables 1**–**4**). Although these methods especially under healthy conditions seem quite similar at first sight, the nature of the cells requires different methodologies. Using the DTA or DTR-system under a cell type specific promoter is a commonly shared and frequently used approach between all glial cell populations. This method has the advantage that it does not require specific features like the expression of a uniquely expressed surface receptor that can be targeted by a drug or being a uniquely mitotically active cell population, but can be applied to all cell types in a similar fashion when used with a specific promoter. The side effects of this method also seem to be rather low and the efficiency quite high, wherefore this method is a very good candidate to be used for future ablation studies of any cell type.

The use of cell specific pharmacological drugs is still an applicable and successful method to ablate cells, but has been so far only exploited for microglia (Elmore et al., 2014; Asai et al., 2015), and astrocytes (Takada et al., 1990; Khurgel et al., 1996). However, although these drugs are supposed to have a very high specificity for a cell type or a surface receptor, there might still be some receptor expression in other cell types, resulting in controversial outcomes of different studies like for instance in the case of the L-AAA-induced astrocyte ablation (Saffran and Crutcher, 1987; Khurgel et al., 1996). The same becomes true for microglia: although there are no controversial reports about the application of PLX3397 to induce myeloid cell death, it is already known that the drug might cross-react with some other receptor-kinases like, e.g., the platelet-derived growth factor receptor (PDGFR) making also other cells expressing those susceptible to cell death (Cornelis et al., 2005; Chitu et al., 2012; Thompson et al., 2015). For NG2-glia there are until now no cell specific drugs available, probably due to the lack of a unique promising target receptor on those cells. In general, the use of cell specific pharmacological drugs still represents an easy and effective way for cell specific ablation studies that does in addition not require the use of expensive transgenic mouse lines. However, the risk of also targeting other cell populations and hence inducing potential secondary effects is still high and the experiments should – as always – be interpreted with caution.

Another very commonly used but also very cell specific approach is the application of X-irradiation to a specific region of interest. But this method can mainly be used for the ablation of NG2-glia, as they carry the unique feature of being the only proliferative cells outside the neurogenic niches, while microglia and astrocytes generally survive this treatment as they do not cycle under physiological conditions (Xu et al., 2007; Buffo et al., 2008). This is in accordance with findings of human X-irradiation studies, where at least low doses of irradiation, do not directly affect the brain but induce “late delayed” effects that do not become evident directly after the treatment but only at later time points, predominantly in the corpus callosum (reviewed in Loganovsky, 2009; Citrin et al., 2010). In this region, the proportion of slowly cycling NG2-glia was shown to be high in humans (Geha et al., 2010) and this region is therefore predominantly affected. However, it has also proven that X-irradiation is not very efficient to ablate NG2-glia, as this method mainly targets actively cycling cells, but NG2-glia were shown to be a very slowly cycling cell population and to have a very long cell cycle due to an extended G1-resting phase (Simon et al., 2011). This unique property is also the reason why infusion of the mitotic blocker AraC specifically ablates NG2-glia (Robins et al., 2013; Djogo et al., 2016).

Most interestingly, although using somehow similar approaches, the ablation efficiencies within the same cell population was highly variable between the different studies (compare **Tables 1**–**4**), especially for oligodendrocytes, where the ablation efficiency spanned from 26 to 80%. This variation could most likely first be explained by the use of different promoters (like MBP or MOG) that are driving the suicide gene expression. Even when using the same promoter, variations in the recombination rate and hence the ablation efficiency can occur, as the cloning strategy of the transgenic animals and the induction protocols can highly influence the recombination rate. The application of the systemic prodrug that can either be given by injections or in the chow would increase the variability between the different studies, as one application form might be more efficient than another. All these arguments do also apply when comparing the area specificity of the ablation approaches that also showed a high variability especially for astrocytes.

After injury, the ablation approaches between at least microglia and astrocytes (as so far no studies have been performed for cells of the oligodendrocyte lineage), are mainly using the TK as a mediator for apoptosis. Again, these studies proved to be quite efficient, both in terms of ablation efficiency and functional readout; might however be accompanied by some disadvantages. TK-mediated cell ablation mainly targets proliferating cells, but not those that are quiescent (Bush et al., 1999), therefore this method does not really display an ablation of the complete cell population but more a paralysis of the injury-driven increase that was observed by *in vivo* imaging studies (Davalos et al., 2005). Especially for astrocytes, this induces a bias for a specific proliferating subset of cells, as only ∼20% of all astrocytes seem to be able to re-enter the cell cycle after injury (Bardehle et al., 2013). Hence, the analysis of the function of another subpopulation of quiescent astrocytes would require another ablation method. For microglia this bias does not seem to be so pronounced, as a higher proportion of microglia is able to proliferate after a pathological insult (Amat et al., 1996), and therefore the function of the whole population seems to be addressed. Thinking forward, this method could be an appropriate approach to ablate also NG2-glia after injury, as 80% of NG2-glia have been shown to proliferate after cortical stab wound injury (Simon et al., 2011).

Brain research generally has the tendency to look at different cell types in a very isolated way, as many of these ablation studies also did, both in the healthy and the pathological brain. In those studies that also determined the consequences of the ablation in other cell types, only the cell numbers were quantified but not their function. However, it is nowadays well accepted that a panglial network exists that is highly connected with each other via connexins (May et al., 2013) and especially after injury seems to also communicate with each other. It is, e.g., known that macrophages after injury are able to influence NG2-glia by secretory mechanisms (Rhodes et al., 2006), or that astrocytes are important for the initiation of the macrophage reactivity (Farina et al., 2007). The ablation of one cell type in the brain could also elicit a reaction in other cell types even when only on the signaling level. Unpublished data from our lab also indicate a cellular communication between the different glial cell types under pathological conditions: when genetically ablating NG2-glia after cortical stab wound injury, the cellular reactions of both astrocytes and microglia was hampered (Schneider and Dimou, unpublished observations), similar to what has already been observed in the spinal cord after a diminished NG2-glia reactivity (Rodriguez et al., 2014).

Taking these new findings into account, the overall sum of glial ablation studies can already provide insights in the cellular characteristics of these cells and help to better understand their function in both the healthy as well as the pathological brain. However, looking to the future, they could be further exploited to investigate the almost unknown terrain of glial cell interactions *in vivo*.

## Author Contributions

SJ structured and wrote the manuscript. LD gave structural and contextual input and corrected the manuscript.

## Conflict of Interest Statement

The authors declare that the research was conducted in the absence of any commercial or financial relationships that could be construed as a potential conflict of interest.
